# The adverse effect of an unplanned surgical excision of foot soft tissue sarcoma

**DOI:** 10.1186/1477-7819-9-160

**Published:** 2011-12-05

**Authors:** Akinobu Nishimura, Akihiko Matsumine, Kunihiro Asanuma, Takao Matsubara, Tomoki Nakamura, Atsumasa Uchida, Ko Kato, Akihiro Sudo

**Affiliations:** 1 Department of Orthopaedic Surgery, Mie University Graduate School of Medicine, 2-174 Edobashi, Tsu City, Mie 514-8507, Japan; 2 Department of Orthopaedics and Sports Medicine Surgery, Mie University Graduate School of Medicine, 2-174 Edobashi, Tsu City, Mie 514-8507, Japan

## Abstract

**Background:**

Malignant soft tissue tumors of the foot are extremely rare and thus can be prematurely excised without appropriate preoperative evaluation. The present study compares adverse effects between unplanned and planned surgical excisions.

**Methods:**

We retrospectively reviewed the clinical records, radiographs, pathology reports and pathological specimens of 14 consecutive patients with soft tissue sarcoma of the foot among 592 with sarcomas between 1973 and 2009. We then compared the incidence and clinical outcomes after unplanned (UT; n = 5) and planned (PT; n = 9) surgical excisions of foot sarcomas.

**Results:**

The most frequent diagnosis was synovial sarcoma (n = 4; 28.6%). The overall 5-year survival rates of the PT and UT groups were 65.6% and 60.0%, respectively, and the event-free 5-year survival rates were 63.5% and 40.0%, respectively. Event-free and overall survival rates did not significantly differ between the two groups. However, tumors were significantly larger in the PT group than in the UT group (p < 0.05).

**Conclusions:**

Unplanned resection lead to a relatively worse prognosis and a likelihood of recurrence despite additional resections. We recommend that soft tumors of the foot should only be excised after appropriate preoperative evaluation regardless of the size of the tumor.

## Background

Soft tissue sarcomas are rare malignancies that account for < 1% of all adult malignancies that annually develop in the United States of America [1]. Among them, malignant soft tissue tumors of the foot are extremely rare, occurring at a rate of 2% to 5% of soft tissue malignancies [2], whereas benign soft-tissue tumors, such as schwannoma, lipomas and hemangiomas, arise more frequently [3-5].

Soft-tissue sarcomas of the foot and benign tumors are notoriously difficult to clinically differentiate [6-8], as their similar presenting features include a palpable mass, swelling, increased warmth, limping and pain. Furthermore, soft-tissue sarcomas of the foot tend to be relatively smaller than those of the proximal limbs, because discomfort while wearing shoes results in seeking medical attention. These non-specific clinical features coupled with the relative rarity and small size, frequently lead to inadequate treatment of soft tissue sarcomas of the foot compared with sarcomas at other anatomical sites [8].

The initial choice of treatment for soft-tissue sarcoma influences the final oncological outcome [6,9]. Thus, wide surgical excision of a sarcoma of the foot after diagnostic imaging, and accurate biopsy is crucial to achieve a good clinical outcome. However, many potential treatment errors are associated with soft tissue sarcomas of the foot. Unplanned surgical excision of a foot sarcoma without appropriate diagnostic evaluation is quite common [6,9,10]. Unplanned surgical excision can result in an inappropriate surgical margin that will require further wide surgical excision and residual disease that can cause repeated local recurrences that can result in limb amputation or be life-threatening.

The present study compares the prognostic outcomes of unplanned and planned surgical excisions of soft-tissue sarcomas of the foot.

## Methods

We retrospectively reviewed the medical records, radiographs and pathology reports of 592 patients who presented at our hospital with soft tissue sarcoma between 1973 and 2009. Among these, we enrolled 14 consecutive patients with foot sarcoma. The exclusion criteria were benign tumors, bone tumors, myeloma, lymphoma, metastatic carcinoma, and tumors arising from the skin. The mean follow-up period was 56.5 months (range 6 - 170 months) after the initial consultation. The patient group included 10 males and four females with a mean age of 35.8 (range 2 - 67) years at the first presentation. Clinical records, radiographs, pathology reports, and pathological specimens were retrospectively reviewed to compare incidences and clinical outcomes of unplanned (UT group) and planned (PT group) surgical resections. All histological diagnoses were made by our institutional pathologists. The tumor grade was assessed according to the updated version of the FNCLCC system based on tumor differentiation, the mitotic count and necrosis [11]. Unplanned surgical excision was defined as that proceeding without preoperative imaging evaluation or staging studies. The cases initially treated by a general surgeon or general orthopedic surgeon without considering the possibility of malignancy were classified in the UT group. Among the UT group patients, extensive hematoma and inappropriate drainage and improper skin incision were observed in 2, 1 and 5 patients, respectively. In contrast, planned surgical resection was defined as that proceeding with appropriate preoperative imaging evaluation. The cases treated by orthopedic oncologists or treated under the supervision of an orthopedic oncologist were classified in the PT group. As a result, there were no patients who showed extensive hematoma, inappropriate drainage or an improper skin incision in the PT group. Variables included patient age, gender, tumor location, grade, surgical margin of initial surgery, tumor size (≥3 cm, group B; < 3 cm, group S), complications, overall survival rates, event-free survival rates and limb preservation (yes or no) between the UT and PT groups. The overall survival was defined as the time from the initial treatment to the date of death attributed to sarcoma. The event-free survival was defined as the time from the initial treatment to the date of clinically documented local recurrence/distant metastasis. The locations of soft-tissue sarcomas in the foot were classified according to the zones described by Kirby et al. [12]. A transverse plane was drawn from the mid-point of the metatarsal heads to the level of the insertion point of the Achilles tendon, and two vertical planes were drawn from the mid-tarsal point to the posterior end of the longitudinal plantar arch and another from the metatarsophalangeal joints down to the sole of the foot. These three lines form five zones, including the region of the ankle (zone 1), heel (zone 2), dorsum of foot (zone 3), sole (zone 4) and toes (zone 5). Soft-tissue sarcomas arising in zone 1 were excluded. Sarcomas were staged according to the Enneking staging system [13].

The Ethics Committees of Mie University approved the study.

### Statistical analysis

Data were statistically analyzed using SPSS Statistics version 18.0 (SPSS Inc, Chicago, IL). P values below 0.05 were considered significant. All data are expressed as means ± standard deviation (SD). Associations in data between the UT and PT groups were determined using an unpaired t-test or the χ^2 ^test. Actuarial data for overall and event-free survival rates at the final follow-up were calculated using Kaplan-Maier analysis.

## Results

Table 1 shows details of the 14 patients who were treated for soft-tissue sarcoma of the foot. The most common diagnoses were synovial sarcoma in 4 patients and clear cell sarcoma and rhabdomyosarcoma in one patient each. Five patients underwent unplanned resection elsewhere before referral (UT group), and nine underwent biopsy and surgical excision at our institution (PT group).

**Table 1:** Patients' data.

Case	Age	Sex	Diagnosis	LC	CC	Stage	FNCLCC grade	Size	FSM	LP	Chem	RT	Follow-up (months)	Prog	Group
1	25	M	RMS	4	Uncertain	II-B	3	S	amp	No	No	Yes	6	DOD	UT
2	46	F	SS	5	Mass	II-B	3	S	amp	No	Yes	No	7	DOD	UT
3	50	M	LPS	3	Mass	I-B	1	B	wide	Yes	No	No	24	CDF	PT
4	2	F	RMS	4	Swelling	III-B	3	S	no op	Yes	Yes	Yes	11	DOD	PT
5	33	M	CCS	5	Mass	II-B	2	B	amp	No	No	No	128	CDF	PT
6	41	M	SS	4	Mass	I-B	2	S	ad	Yes	No	No	89	CDF	UT
7	10	M	FS	3	Mass	II-B	2	B	wide	Yes	Yes	No	170	CDF	PT
8	67	M	CSSP	4	Uncertain	II-B	2	B	amp	No	No	No	7	DOC	PT
9	42	M	LMS	4	Pain, mass	II-B	2	B	amp	No	Yes	No	38	DOD	PT
10	39	F	SS	4	Tenderness	II-B	2	B	ad	Yes	Yes	No	92	AWD	UT
11	47	M	SS	4	Mass	II-B	3	B	amp	No	No	No	110	CDF	PT
12	19	F	CCS	2	Tenderness	II-B	2	S	ad	Yes	No	Yes	84	NED	UT
13	66	M	ESMCS	4	Uncertain	I-B	1	B	wide	Yes	No	Yes	12	AWD	PT
14	14	M	DFSP	3	Mass	II-A	2	B	wide	Yes	No	No	13	CDF	PT

Patients are in chronological order. LC, location; CC, chief complaint; FNCLCC grade, Fédération Nationale des Centres de Lutte Contre le Cancer Grading System; FSM, final surgical margin; LP, limb preservation; Chem, chemotherapy; RT, radiation therapy; Prog, Prognosis. CCS, Clear cell sarcoma; CSSP, chondrosarcoma of soft part; DFSP, dermatofibrosarcoma protuberance; ESMCS, extraskeletal myxoid chondrosarcoma; FS, fibrosarcoma; LMS, leiomyosarcoma; LPS, liposarocoma; RMS, rhabdomyosarcoma; SS, synovial sarcoma. Tumor size: S, < 3 cm; B, ≥3 cm; amp, amputation; wide, wide resection; no op, no operation; ad, additional wider resection; AWD, alive with disease; CDF, complete disease free; NED, no evidence of disease; DOD, died of disease; DOC, died of other causes. PT, planned excision; UT, unplanned excision.

Seven patients had wide excision of the tumors or additional wider excision of lesions that had been inadequately excised. Six patients had undergone amputation. One patient died of the disease before undergoing wide resection. Another patient did not require additional excision because the estimated surgical margin after the initial excision was negative. Four patients died due to the sarcoma and all of them had pulmonary metastasis. One patient died of other causes. The mean time from surgery to death was 13.8 months (range 11 - 38).

Chemotherapy was administered to 5 patients with synovial sarcoma, rhabdomyosarcoma, fibrosarcoma and leiomyosarcoma. Radiation therapy was administered to 2 patients. Only one patient with high-grade sarcoma (rhabdomyosarcoma) received both chemotherapy and radiation therapy. Five did not receive adjuvant chemotherapy after resection because the histological grade was low or intermediate.

**Figure 1:**
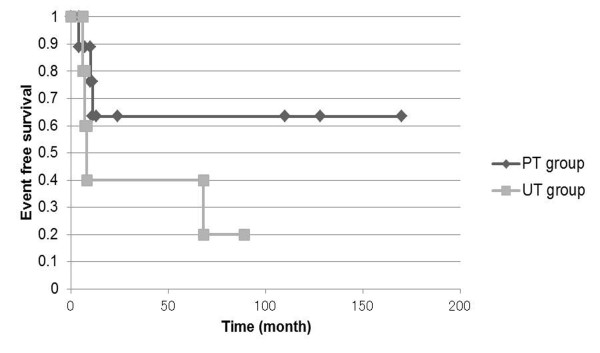
**Kaplan-Meier overall survival estimates for unplanned and planned excisions of foot sarcoma**. No significant differences between PT and UT groups. PT, planned excision; UT, unplanned excision.

**Figure 2:**
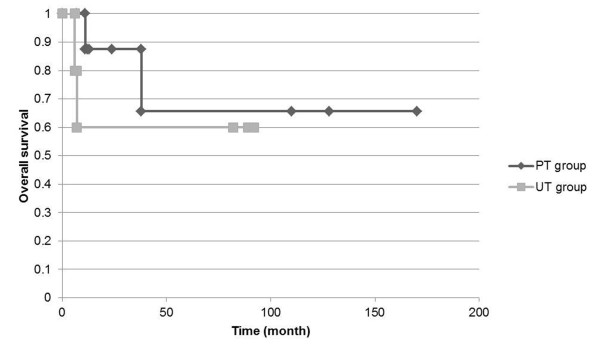
**Kaplan-Meier event-free survival estimates for unplanned and planned excisions of foot sarcoma**. No significant differences between PT and UT groups. PT, planned excision; UT, unplanned excision.

Figures 1 and 2 show Kaplan-Meier analysis for event-free and overall survival rates, respectively. The overall 5-year survival rate of all patients was 66.8%. The overall 5-year survival rates for the PT and UT groups were 65.6% and 60.0%, respectively. The overall event-free 5-year survival rate of all of our patients was 55.0%. The event-free 5-year survival rates for the PT and UT groups were 63.5% and 40.0%, respectively. Two developed local recurrence and six developed lung metastases. Only one patient with clear cell sarcoma had inguinal node metastases. Event-free and overall survival rates did not significantly differ between the PT and UT groups.

Among patient age, gender, tumor location, histological grade, tumor size, complications and limb preservation, only tumor size significantly differed between the UT and PT groups (p < 0.05).

## Discussion

Soft tissue tumors or tumor-like lesion of the foot are usually histologically benign [4]. Ganglions comprise about one-third of all soft tissue tumors [5]. Because patients with asymptomatic small tumors do not often receive appropriate medical care, the literature does not always reflect the true proportion of soft-tissue tumors that are foot sarcomas. However, Berlin [3] identified 449 sarcomas in an analysis of 307,601 tumors and tumor-like conditions of the foot. Kirby et al. [4] reviewed 83 patients with soft-tissue tumors and tumor-like conditions of the foot and ankle and identified 11 (13%) malignant tumors of which synovial sarcoma was the most frequent. Temple et al. [8] described 12 (34.3%) synovial sarcomas among 39 soft tissue sarcomas of the foot and ankle. Thacker et al. [14] described 17 (32.7%) among 52 soft tissue sarcomas and Cribb et al. [15] found 12 (44.4%) among 27 soft tissue sarcomas. We found here that synovial sarcomas (n = 4; 28.6%) comprised the most frequent soft tissue sarcoma of the foot, even after tumors originating in the ankle were excluded.

The prognosis of sarcomas is better when they are located in the distal extremity compared with the proximal extremity. Zeytoonjian et al. [16] described that the overall death rate all sarcomas was 26.6% (630/2367 patients), whereas that of foot sarcoma was 10.3% (18/170 patients). Cribb et al. [15] reported a rate of 14.8% (4/27 patients). Thacker et al. [14] indicated a 5-year survival rate of 78.2% (11/52 patients) among 52 patients. The 5-year survival rate of 66.8% (4/14 patients) in the present study was relatively lower than those described by others [14-16]. However, the 5-year survival rate of latest five patients was 100%. We speculated that improvements in diagnostic imaging technology including MRI enabled accurate preoperative evaluation of tumors. MRI is useful to know tumor size, tumor location and tumor margin. And, we believe that preoperative MRI is useful for evaluating and diagnosing foot tumors, because most of the benign soft-tissue tumors that frequently occur on the foot, such as schwannoma, epidermal cysts, ganglion cysts, lipomas and hemangiomas, can be diagnosed using MR imaging [3-5]. For the diagnosis of schwannoma, Tinel's-like sign with specific MRI findings (target sign) may be useful. In the case of epidermal cysts, T1-weighted images after gadolinium administration show rim-enhancement with subcutaneous involvement. Ganglion cysts can be easily diagnosed because of the homogenous intensity of the lesion. Lipomas can also be easily diagnosed due to their homogeneous high-intensity area on both T1-and T2- weighted images. Although hemangioma is sometimes difficult to distinguish from malignancy, the MR findings often show lobulation, septation, and central low-signal-intensity dots with small fat intensity foci. On the contrary, when a definite imaging diagnosis can not be obtained, the surgeon should carefully excise the tumor after considering the possibility of malignancy, even if the tumor size is small. These preoperative imaging analyses might have been associated with a good clinical result.

Five (35.7%) of 14 patients in the present study had unplanned excisions. However, Davis et al. [6] and Goodlad et al. [10] reported that 43.5% and 40% of patients with soft-tissue sarcomas in their series, respectively, underwent unplanned resections; our findings were comparable to these results. The physical features of soft tissue sarcomas of the foot, including palpable mass, swelling, local warmth, and pain, are similar to those of benign lesions, and thus many smaller lesions tend to undergo simple unplanned excisions without appropriate diagnostic imaging and biopsies. These non-specific features might in part facilitate the high incidence of unplanned surgical excisions. Indeed, tumors were significantly smaller in the UT group than in the PT group in the present series. This result might paradoxically show that smaller tumors throw clinicians off guard. An excisional biopsy may be justified when the tumor is less than 3 cm. However, the problem is whether the initial surgery was performed while considering the possibility of malignancy. Unfortunately, in this study, all of the UT group patients were initially treated without considering the possibility of malignancy, and this led to extensive hematoma formation, inappropriate drainage and improper skin incisions. Therefore, we classified the cases initially treated by general surgeons or general orthopaedic surgeon without considering the possibility of malignancy as part of the UT group.

Unplanned excisions of soft tissue sarcomas are frequent, but their influence on local recurrence, distant metastasis and patient survival remains controversial [17-19]. Davis et al. [6] compared the clinical outcomes between patients primarily treated at an institution specializing in cancer and those who were referred following unplanned excision at a general hospital. They found a higher local recurrence rate in the group with unplanned surgical excisions, especially those with residual tumors in re-resected specimens. Clasby et al. [9] reviewed 377 patients with primary soft-tissue sarcomas, and estimated that 21.3% (80/377 patients) of them had been inadequately treated. Furthermore, they noted a poorer outcome among patients with tumors that recurred after marginal excision. Thacker et al. [14] reported 5-year oncological survival rates of 80.4% (21/23 patients) and 73.4% (22/29 patients) in patients who underwent planned and unplanned excisions, respectively, and 5-year event free survival rates of 68.1% (9/23 patients) and 60.8% (11/23 patients), respectively. Temple et al. [8] reported 4-year oncologic survival rates of 76.5% (13/17 patients) and 77.8% (12/18 patients) in the PT and UT groups, respectively, and local recurrence rates of 11.8% (2/17 patients) and 16.7% (3/18 patients), respectively.

In the present series, 5-year overall survival rate in the PT group was 65.6% (7/9 patients) and that in the UT group was 60.0% (3/5 patients). The 5-year event free survival rates were 63.5% (6/9 patients) and 40.0% (2/5 patients) in the PT and UT groups, respectively. Rates of tumor recurrence or tumor death did not significantly differ between the UT and PT groups. However, tumors were significantly larger in the PT group than in the UT group in this study. Larger tumors significantly and negatively affect prognosis in patients with soft tissue sarcoma [20,21]. These findings suggested a relatively worse prognosis for the UT group than for the PT group. Because the absence of a statistical difference was probably due to the low number of recurrences and metastasis in the present study, further large-scale studies are warranted.

In point of the anatomical site of foot sarcoma, dorsal side (location 3) sarcomas of the foot were all big size (> 3 cm), On the other side, plantar side (loation 2 and 4) sarcomas of the foot were 4/9 cases small size and 5/9 big size. Thus, dorsal side sarcomas of the foot tend to be neglect until the tumor grew big size. Patients who have plantal sarcomas of the foot tend to be seen in hospital earlier, because they might feel discomfort and pain of their sole.

The major shortcoming of this study was the limited patient population, because soft tissue sarcoma of the foot is extremely rare. Therefore, the statistical power of this study is very low.

Most soft tissue tumors of the foot are small and benign, which results in frequent unplanned excisions. Although benign tumors would not pose a clinical problem, malignant tumors lead to a higher incidence of local recurrence even after additional surgery and result in a poor prognosis. We emphasize that unplanned excisions should be averted by appropriate preoperative evaluation such as by MRI, early referral of all potential malignancies to a cancer center, biopsy assessment and wide excision of malignant lesions.

## Conclusions

Sarcomas of the foot are rare and thus tend not to be suspected by either patients or clinicians. Event-free and overall survival rates did not significantly differ between the PT and UT groups, but tumors were significantly larger in the PT group. Larger tumors negatively affect the prognosis in patients with soft tissue sarcoma. Therefore, we concluded that the UT group had a relatively worse prognosis than the PT group. Therefore, we conclude that unplanned resection leads to a relatively worse prognosis and a likelihood of recurrence despite additional resections. We recommend that even small soft tumors of the foot should be preoperatively evaluated by imaging to avoid repeated local recurrence.

## Competing interests

The authors declare that they have no competing interests.

## Authors' contributions

AM was the lead author and surgeon for all of the patients. KA, TM and TN were the co-surgeon on the cases and contributed patients and information on the patients. AU reviewed paper and technique of surgery. KK contributed to writing of the paper. AS reviewed paper. All authors read and approved the final manuscript.
